# 色谱柱选择对23种防腐剂测定结果的影响

**DOI:** 10.3724/SP.J.1123.2021.05006

**Published:** 2022-02-08

**Authors:** Li LI, Shuo LI, Haiyan WANG, Lei SUN

**Affiliations:** 中国食品药品检定研究院, 北京 100050; National Institutes for Food and Drug Control, Beijing 100050, China; 中国食品药品检定研究院, 北京 100050; National Institutes for Food and Drug Control, Beijing 100050, China; 中国食品药品检定研究院, 北京 100050; National Institutes for Food and Drug Control, Beijing 100050, China; 中国食品药品检定研究院, 北京 100050; National Institutes for Food and Drug Control, Beijing 100050, China

**Keywords:** 高效液相色谱, 色谱柱选择, 关键因素, 防腐剂, 化妆品, high performance liquid chromatography (HPLC), chromatographic column selection, key factors, preservatives, cosmetics

## Abstract

以化妆品中23种防腐剂检测方法为例,探讨色谱柱选择对液相色谱方法测定结果的影响。参照《化妆品安全技术规范》甲基异噻唑啉酮等23个组分的检验方法,在2台不同的高效液相色谱仪上用15款不同品牌、型号的C_18_色谱柱检测23种防腐剂,计算色谱峰的理论塔板数和分离度,对23种组分的分离效果进行分析,并应用USP (United States Pharmacopeia)数据库和PQRI (Product Quality Research Institute)数据库等2种等效色谱柱选择方法,对不同色谱柱的分离效果及等效性进行评价和预测。实验结果表明,15款色谱柱对23种防腐剂的分离效果差异显著,仅有2款色谱柱可以实现23种组分的完全分离。USP和PQRI数据库中2种等效色谱柱选择方法均无法预测出合适的等效色谱柱,对23种防腐剂的液相色谱分析参考价值均较小。色谱柱是影响23种防腐剂液相色谱法测定结果准确性的关键因素,有关实验室在应用该方法时,应考虑色谱柱选择性差异。化妆品基质复杂,如何在现有研究成果的基础上,开发色谱柱的筛选和预测评价体系,进而指导实际样品的分离是下一步研究的重点、难点。建议有关部门在制修订检测方法时,注重色谱柱的耐用性考察,完善系统适应性指标,细化色谱柱分类和增加描述信息,指导色谱柱的合理选择,从而规避由于色谱柱使用过程中选择依据缺失而导致测定结果不准确的风险。

## Abstract (en)

The influence of column selection on the determination results of 23 preservatives in cosmetics was studied by considering the corresponding detection methods for these preservatives. The test method for 23 preservatives specified in *Safety and Technical Standards for Cosmetics* was considered as the reference method. Twenty three preservatives were analyzed using 15 different brands and models of C_18_ chromatographic columns on two different high performance liquid chromatographs. The plate count and resolution of the chromatographic peaks were calculated to analyze the separation effect of the 23 components. The separation efficiency and equivalence of different chromatographic columns were evaluated and predicted using the database established by the United States Pharmacopeia (USP) and Product Quality Research Institute (PQRI). The experimental results showed that the separation effects of the 15 chromatographic columns on the 23 preservatives differed significantly. Further, only two chromatographic columns were able to completely separate the 23 components without changing the standard procedure for mobile phase elution and other conditions. The other 13 columns poorly separated some components. The retention times of phenyl 4-hydroxybenzoate, isobutyl 4-hydroxybenzoate, butyl 4-hydroxybenzoate, and benzyl 4-hydroxybenzoate were similar. The separation of these substances was among the key outcomes of the chromatographic separation. The differences in the selectivity of the 4-hydroxybenzoates were distinct among the different chromatographic columns. The plate counts of the 4-hydroxybenzoates were similar when using the same chromatographic column on two high performance liquid chromatographs. However, the plate counts of 4-hydroxybenzoates using different chromatographic columns were considerably different. Control of the plate counts alone was not sufficient to achieve good separation efficiency of the 4-hydroxybenzoates. Moreover, the peak sequences of some compounds such as 4-hydroxybenzoic acid and methylisothiazolinone with different chromatographic columns were reversed. Some substances could be absorbed at multiple wavelengths. If separation from adjacent chromatographic peaks is not achieved at the baseline, large errors will occur when detecting the low-concentration components to be tested. The ZORBAX SB-C_18_ column was used as a reference column, and the similarity values provided by the USP and the PQRI databases were consulted. The actual separation efficiency of the typical column used in the experiment was compared with the predicted results of selectivity difference from the database. The results showed that the USP and the PQRI database could not predict a suitable equivalent column. There was no regularity in the experimental results; further, the USP and the PQRI datebase provided scarce reference values for the analysis of the 23 preservatives by liquid chromatography. The outcomes obtained using liquid chromatography methods are easily affected by factors such as the instrument model, mobile phase composition and pH, chromatographic column, column temperature, and flow rate. The chromatographic column is a key factor affecting the accuracy of determination by HPLC. The selectivity difference of the chromatographic column should be taken into account when the technique is performed in relevant laboratories. The standard technique will be employed by different laboratories at different times and under different test conditions, and many abnormalities or deviations may occur. The sequence of peaks should be confirmed using a single compound standard during the first standard method validation or during the replacement of columns. Based on existing research results, future research should focus on the development of a screening and prediction evaluation system for chromatographic columns, and thereby guide the separation of actual samples in complex cosmetic bases. Relevant laboratories should focus on the durability of chromatographic columns, and improve the system adaptability index when developing a detection method. They also should refine the column classification and enhance descriptive information, and thus, guide the rational selection of the column so as to mitigate the risk posed by inaccurate determination results due to improper selection of the chromatographic column.

为避免化妆品在生产、储存和使用的过程中受到微生物污染,延长产品保存期限和保障消费者使用安全,生产厂家通常在化妆品配方中添加一种或多种防腐剂^[1]^。目前化妆品准用防腐剂共有51种,《化妆品安全技术规范》仅对单一物质的最大允许使用量进行了限定,但未对单一化妆品中可以使用的防腐剂种类数量做出规定,也未对产品中多种防腐剂混合使用的最大使用量进行限定。市售化妆品中多种防腐剂配合使用的情况较为普遍,加入多种防腐剂不但可以扩大抗菌广谱性,起到较好的抑菌作用,同时还可以降低每种防腐剂的用量,避免超出限量^[2]^。防腐剂需要达到一定的使用浓度才能发挥其抑菌的功效,因此添加量一般在千分比到百分比的级别^[2,3]^。

目前,化妆品中防腐剂的检测方法主要有高效液相色谱法、液相色谱-质谱法、气相色谱法、气相色谱-质谱法等^[3,4,5,6,7]^。高效液相色谱法灵敏、高效,选择性强,而且51种准用防腐剂中大部分组分都能用该法分析,所以该技术已经成为应用最广泛的检测方法。色谱柱是高效液相分离系统中最重要的环节,色谱柱的类型决定色谱系统的性质,色谱柱的粒径和长度影响分析时间和分析效率^[8]^,采用不同品牌、不同型号的色谱柱分析具体样品时,由于色谱柱硅胶纯度、键合工艺和封尾技术等工艺方面的不同造成选择性差异较大,溶质的保留值、色谱峰之间的分离度甚至色谱峰顺序都可能出现较大的差异^[9,10,11]^。特别是当样品基质中含碱性或弱酸性化合物时这种风险更为显著^[9]^。如果使用的色谱柱不合适,目标物色谱峰和干扰峰之间分离度则达不到要求,从而影响定性和定量结果的准确性。因此,在实际检验工作中,尤其是进行高效液相色谱法分析时,色谱柱的选择是一项十分重要且困难的工作,关系到整个实验的成败^[12]^。我国《化妆品安全技术规范》理化检验方法在描述高效液相色谱方法时,通常仅列出色谱柱的填料种类、孔径、颗粒大小等基本信息,如“C_18_柱(250 mm×4.6 mm, 5 μm)或等效色谱柱”,为了避免商业竞争,在非必要条件下一般不予公开方法使用的色谱柱品牌、型号等具体信息。虽然这样做的初衷是为了体现方法的公平性,但也在一定程度上增加了检验方法实际执行的难度,特别是多种组分同时分析时,色谱柱的选择存在一定的盲目性。

自20世纪70年代开始,国内外不少研究人员开始研究影响色谱柱选择性的因素,尝试建立量化表征方法,用于比较不同品牌型号色谱柱的选择性差异。根据色谱柱填料的不同将C_18_色谱柱分为3类^[13]^: A型色谱柱(早期开发的色谱柱,硅胶纯度较低,硅羟基和金属残留较高)、B型色谱柱(新型色谱柱,硅胶纯度较高,金属残留量低,是目前市场上主流色谱柱)、E型色谱柱(C_18_链内嵌极性基团或极性基团封尾的色谱柱)。但这种分类模式比较笼统,与色谱柱的选择性缺乏关联,难以表征色谱柱选择性的差异^[10]^。21世纪初,研究者通过采用不同的色谱柱参数和化学计量学方法,逐渐建立色谱选择性的柱表征体系^[10,11]^。目前比较有影响力的色谱柱数据库包括^[9]^: USP(United States Pharmacopeia)、PQRI(Product Quality Research Institute)、KUL(Katholieke Universiteit Leuven)和ACD(Advanced Chemistry Development)数据库,每个数据库都包含了数百种色谱柱信息,但以上4个数据库均未收载国产色谱柱的信息。化妆品种类繁多,成分复杂,如何对多组分进行有效分离一直是化妆品分析检测的难点,本研究以《化妆品安全技术规范》收载的23种防腐剂的测定方法为例,探讨色谱柱对高效液相色谱分析结果的影响,并结合现有的色谱柱研究成果和评价手段,尝试对不同色谱柱针对化妆品23种防腐剂的选择性及等效性进行评价和预测,以期为化妆品理化检验中该类难题的解决提供研究思路和数据支持。

## 1 实验部分

### 1.1 仪器、试剂与材料

Waters e2695高效液相色谱仪,配二极管阵列检测器,美国Waters公司;Agilent 1260高效液相色谱仪,配二极管阵列检测器,美国Agilent公司;XP205电子天平,瑞士Mettler-Toledo公司。C_18_色谱柱规格、型号、厂家等参数见表1。

**表 1:** 色谱柱信息

No.	Brand	Model	Specification	pH range		Category	Status
1	Osaka Soda	Capcell Pak MG Ⅲ C_18_	250 mm×4.6 mm, 5 μm	2-	10	B	used
2	Osaka Soda	Capcell Pak MG Ⅱ C_18_	250 mm×4.6 mm, 5 μm	2-	10	B	used
3	Osaka Soda	Capcell Pak MG C_18_	250 mm×4.6 mm, 5 μm	2-	10	B	used
4	Agilent	ZORBAX SB-C_18_	250 mm×4.6 mm, 5 μm	1-	8	B	brand new
5	Agilent	ZORBAX Extend-C_18_	250 mm×4.6 mm, 5 μm	2-	11.5	B	used
6	Waters	Xbridge C_18_	250 mm×4.6 mm, 5 μm	1-	12	B	used
7	Waters	Xselect T_3_	250 mm×4.6 mm, 5 μm	2-	8	B	brand new
8	Waters	Xbridge Shield RP C_18_	250 mm×4.6 mm, 5 μm	2-	11	E	brand new
9	Akzo Nobel	Kromasil 100-5C_18_	250 mm×4.6 mm, 5 μm	1.5-	10	B	used
10	Thermo	BDS HYPERSIL C_18_	250 mm×4.6 mm, 5 μm	2-	8	A	used
11	Phenomenex	LUNA C_18_(2)	250 mm×4.6 mm, 5 μm	1.5-	10	B	brand new
12	Gl Sciences	InertSustain AQ-C_18_	250 mm×4.6 mm, 5 μm	1-	10	B	brand new
13	Acchrom	Unitary C_18_	250 mm×4.6 mm, 5 μm	2-	8	B	brand new
14	Dikma	DIAMONSIL C_18_	250 mm×4.6 mm, 5 μm	2-	9	B	used
15	Agela	INNOVAL C_18_	250 mm×4.6 mm, 5 μm	1.5-	9	B	brand new

A: old-type columns; B: new-type columns; E: embed-depolar group columns.

23种防腐剂标准品及其纯度信息见表2。甲醇、乙腈、二甲亚砜均为色谱纯,购自德国Merck公司;磷酸为分析纯,购自国药集团化学试剂有限公司。

### 1.2 实验方法

参照《国家药监局关于将化妆品中防腐剂检验方法等7项检验方法纳入化妆品安全技术规范(2015年版)的通告》(2021年第17号)新发布的《化妆品安全技术规范》第4章4.1节甲基异噻唑啉酮等23个组分的检验方法(该方法以下简称为“规范方法”)对化妆品中23种防腐剂进行测定。

1.2.1 标准溶液的配制

混合标准储备溶液:准确称取23种标准品适量(精确至0.00001 g),置于同一容量瓶中,先用2 mL二甲亚砜溶解,再用乙腈定容至刻度,摇匀,置于4 ℃冰箱中冷藏保存。

混合标准工作溶液:取混合标准储备溶液适量,用甲醇稀释得混合标准工作溶液,现用现配。

1.2.2 液相色谱分析条件

色谱柱:C_18_色谱柱(250 mm×4.6 mm, 5 μm);柱温:30 ℃;流动相A:磷酸水溶液(量取磷酸1.2 mL,加水至1000 mL,混匀);流动相B:乙腈;流速:1.0 mL/min;梯度洗脱程序:0~5 min, 10%B; 5~29 min, 10%B~50%B; 29~41 min, 50%B ~35%B; 41~52 min, 35%B~65%B; 52~57 min, 65%B ~95%B; 57~62 min, 95%B~10%B;进样量:10 μL。检测波长(*λ*)信息见表2。

**表 2:** 23种防腐剂的纯度和检测波长

No.	Compound	Chinese name	Purity/%	λ/nm
1	methylisothiazolinone	甲基异噻唑啉酮	98.6	280
2	bronopol	2-溴-2-硝基丙烷-1,3-二醇	99.4	230
3	4-hydroxybenzoic acid	4-羟基苯甲酸	99.9	254
4	methylchloroisothiazolinone	甲基氯异噻唑啉酮	99.3	280
5	benzylalcohol	苯甲醇	99.8	254
6	2-phenoxyethanol	苯氧乙醇	99.8	280
7	benzoic acid	苯甲酸	99.9	230
8	methyl 4-hydroxybenzoate	4-羟基苯甲酸甲酯	99.7	254
9	chlorphenesin	氯苯甘醚	99.3	230
10	dehydroacetic acid	脱氢乙酸	99.9	280
11	5-bromo-5-nitro-1,3-dioxane	5-溴-5-硝基-1,3-二噁烷	99.7	230
12	ethyl 4-hydroxybenzoate	4-羟基苯甲酸乙酯	99.0	254
13	4-hydroxybenzoic acid isopropyl ester	4-羟基苯甲酸异丙酯	99.0	254
14	propyl 4-hydroxybenzoate	4-羟基苯甲酸丙酯	98.7	254
15	phenyl 4-hydroxybenzoate	4-羟基苯甲酸苯酯	99.0	254
16	4-hydroxybenzoic acid isobutyl ester	4-羟基苯甲酸异丁酯	99.6	254
17	n-butyl 4-hydroxybenzoate	4-羟基苯甲酸丁酯	99.9	254
18	benzyl 4-hydroxybenzoate	4-羟基苯甲酸苄酯	99.5	254
19	ethyl benzoate	苯甲酸乙酯	99.8	230
20	amyl 4-hydroxybenzoate	4-羟基苯甲酸戊酯	99.8	254
21	isopropyl benzoate	苯甲酸异丙酯	99.8	230
22	propyl benzoate	苯甲酸丙酯	99.5	230
23	phenyl benzoate	苯甲酸苯基酯	99.9	230

1.2.3 样品测定

取混合标准工作溶液按照1.2.2节液相色谱条件,用表1中的15款色谱柱在Waters e2695高效液相色谱仪和Agilent 1260高效液相色谱仪上分别进行测定。记录23种组分的保留时间、出峰顺序等色谱柱的保留行为,并计算分离度(*R*)和理论塔板数(*n*)。

## 2 结果与讨论

### 2.1 不同色谱柱的分离效果

在Waters e2695液相色谱仪上按照1.2.2节条件,分别用15款色谱柱对23种防腐剂组分进行测试,从部分有代表性的色谱图可以看出,不同品牌型号C_18_柱的分离效果存在明显差异(见图1)。经过对比发现,在不改变规范方法流动相洗脱程序等其他条件的前提下,15款色谱柱中只有2款色谱柱(Capcell Pak MG Ⅲ C_18_和ZORBAX SB-C_18_)能够将23种组分完全分离,其他13款色谱柱都存在部分组分分离度不佳的情况。

**图1:**
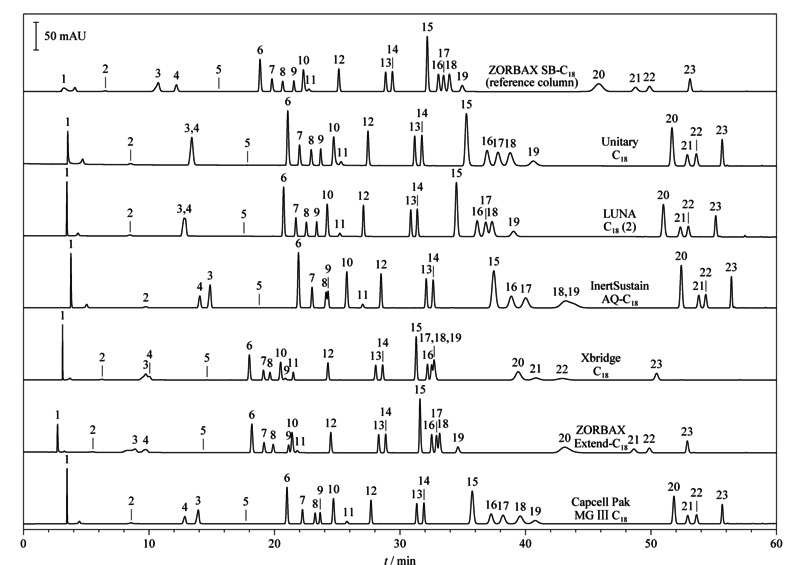
采用不同色谱柱在Waters e2695液相色谱仪上检测23种防腐剂的色谱图(*λ*=280 nm)

### 2.2 不同色谱柱对同类物质的分离情况

4-羟基苯甲酸苯酯、4-羟基苯甲酸异丁酯、4-羟基苯甲酸丁酯、4-羟基苯甲酸苄酯等同类物质及同分异构体保留时间相近,是色谱分离的关键点之一。色谱柱的分离效能最重要的评价指标为分离度和理论板数。选取4-羟基苯甲酸丁酯为代表组分计算理论板数和其与相邻色谱峰(前峰为4-羟基苯甲酸异丁酯,后峰为4-羟基苯甲酸苄酯)的分离度*R*_1_和*R*_2_(*λ*=254 nm),结果见表3。可以看出,同一色谱柱在2台仪器上的理论塔板数相近,不同色谱柱之间的理论板数差异较大,但理论板数和4-羟基苯甲酸酯类物质的分离效果不成正相关;15款色谱柱中除5款色谱柱对于4-羟基苯甲酸丁酯和4-羟基苯甲酸异丁酯这组同分异构体的分离效果稍差外,其余11款色谱柱都能实现基线分离(分离度≥1.5)。但是对于4-羟基苯甲酸丁酯和4-羟基苯甲酸苄酯,仅有6款色谱柱能实现良好分离。不同色谱柱对4-羟基苯甲酸酯类的保留行为选择性差异较大,仅控制色谱柱柱效并不能保证该类物质的分离效果。

**表 3:** 4-羟基苯甲酸丁酯在不同色谱柱上的理论塔板数及与相邻峰的分离度(*λ*=254 nm)

No.	Column	Agilent 1260 HPLC				Waters e2695 HPLC		
		n	R_1_	R_2_		n	R_1_	R_2_
1	Capcell Pak MG Ⅲ C_18_	74930	1.7	1.7		65464	1.7	2.0
2	Capcell Pak MG Ⅱ C_18_	68190	1.4	1.4		81147	1.4	1.3
3	Capcell Pak MG C_18_	97912	1.6	1.7		138635	1.6	1.7
4	ZORBAX SB-C_18_	243033	1.5	1.6		273043	1.6	1.6
5	ZORBAX Extend-C_18_	296705	1.6	1.0		331689	1.6	1.0
6	Xbridge C_18_	212670	1.3	0.7		146192	0.7	0.5
7	Xselect T_3_	89553	1.3	1.7		108543	1.3	1.7
8	Xbridge Shield RP C_18_	173712	1.7	2.4		206534	1.8	2.4
9	Kromasil 100-5C_18_	80564	1.7	0.8		74783	1.7	1.3
10	BDS HYPERSIL C_18_	303155	1.5	0.8		276142	1.4	0.8
11	LUNA C_18_(2)	77920	1.5	1.0		95581	1.5	1.0
12	InertSustain AQ-C_18_	31145	1.6	3.5		40402	1.6	2.8
13	Unitary C_18_	57033	1.6	1.6		60985	1.5	1.5
14	DIAMONSIL C_18_	39073	1.3	1.1		21805	1.2	1.9
15	INNOVAL C_18_	353918	1.6	1.1		400896	1.7	1.1

R_1_: resolution of n-butyl 4-hydroxybenzoate and 4-hydroxybenzoic acid isobutyl ester; R_2_: resolution of n-butyl 4-hydroxybenzoate and benzyl 4-hydroxybenzoate.

### 2.3 不同色谱柱对不同类物质的分离情况

选取对4-羟基苯甲酸苯酯、4-羟基苯甲酸异丁酯、4-羟基苯甲酸丁酯、4-羟基苯甲酸苄酯等物质分离效果较好的5款色谱柱,考察不同色谱柱对4-羟基苯甲酸甲酯、氯苯甘醚、脱氢乙酸、5-溴-5-硝基-1,3-二噁烷的分离情况。计算用Waters e2695液相色谱仪检测时,目标组分与相邻色谱峰的*R*值,结果见表4。可以看出,4-羟基苯甲酸甲酯、氯苯甘醚、脱氢乙酸、5-溴-5-硝基-1,3-二噁烷虽然结构差异较大,检测波长也不同,但在C_18_色谱柱上保留行为相似,色谱峰的保留时间相近。这4种化合物在5款色谱柱上的分离度存在明显差异,4-羟基苯甲酸甲酯和氯苯甘醚在InertSustain AQ-C_18_色谱柱上没有完全分离。虽然4种化合物采用不同的检测波长检测一定程度上减少了色谱峰分离度不足对定量结果带来的影响,但是由于部分物质在多个波长下均有吸收,如果与相邻色谱峰不能实现基线分离,对低浓度待测组分检测时仍会引起较大误差。

**表 4:** 4种化合物在5款色谱柱上的保留时间和分离度

Compound	λ/nm	Capcell Pak MG Ⅲ C_18_			Capcell Pak MG C_18_			ZORBAX SB-C_18_			InertSustain AQ-C_18_			Unitary C_18_		
		t_R_/min	R		t_R_/min	R		t_R_/min	R		t_R_/min	R		t_R_/min		R
Methyl 4-hydroxybenzoate	254	23.24	4.9		22.53	4.3		20.65	3.7		24.1	4.9		22.93		4.1
Chlorphenesin	230	23.64	2.1		23.20	4.7		21.54	4.2		24.3	1.0		23.68		3.6
Dehydroacetic acid	280	24.70	5.1		23.52	1.5		22.30	3.3		25.7	6.4		24.72		4.3
5-Bromo-5-nitro-1,3-dioxane	230	25.77	4.2		24.06	2.0		22.74	1.5		27.0	4.8		25.30		1.8

### 2.4 不同色谱柱对色谱峰出峰顺序的影响

不同色谱柱对23种防腐剂的保留行为差异除了体现在分离度上之外,在色谱峰的出峰顺序上也存在显著不同。实验结果表明,在用Waters e2695液相色谱仪检测时,4-羟基苯甲酸(图2中3号峰)和甲基氯异噻唑啉酮(图2中4号峰)在7款色谱柱(DIAMONSIL C_18_、InertSustain AQ-C_18_、Xbridge Shield RP C_18_、Xselect T_3_、Capcell Pak MG C_18_、Capcell Pak MG Ⅱ C_18_和Capcell Pak MG Ⅲ C_18_)上的出峰顺序和规范方法给出的参考色谱图出峰顺序相反。4-羟基苯甲酸和甲基氯异噻唑啉酮在Unitary C_18_和LUNA C_18_(2)这2款色谱柱上的色谱峰完全重叠,没有分开(见图2)。

**图2:**
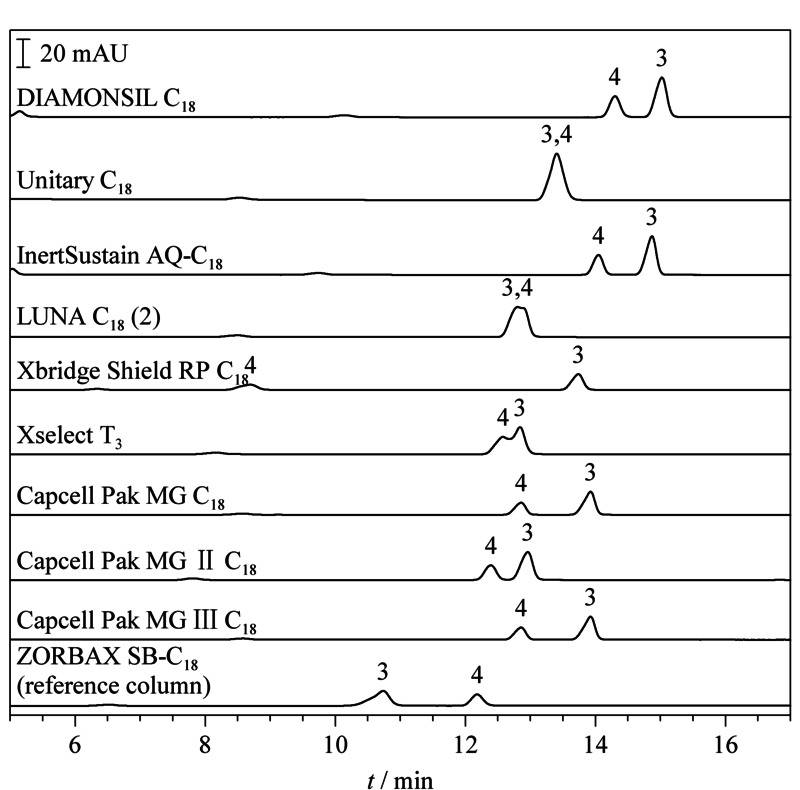
采用不同色谱柱在Waters e2695液相色谱仪上检测时4-羟基苯甲酸和甲基氯异噻唑啉酮的色谱图(*λ*=280 nm)

对于化妆品中多组分检测项目,实际工作中往往需要配制十几种或几十种标准溶液,费时费力,极大制约了工作效率,因此一些试剂公司推出了按照规范方法预先配制好的混合标准溶液等产品。检验人员在使用这些产品给工作带来便捷的同时,还应注意由于色谱柱选择不合适带来的误判问题。在标准方法或规范方法首次验证或更换色谱柱检测时,都应当使用单一化合物的标准品对色谱峰的出峰顺序进行确证。

### 2.5 等效色谱柱选择系统的应用

USP网站(https://apps.usp.org/app/USPNF/columnsDB.html)提供USP和PQRI数据库中2种色谱柱相似度计算和预测工具。选择参比色谱柱后,可获得其他色谱柱与参比色谱柱相似度值(*F*), *F*越小,说明2款色谱柱的选择性越相似,当*F*≤3时,认为2款色谱柱的选择性相似,可视为等效色谱柱。以规范方法制定单位在该方法编制说明中给出的色谱柱(Agilent ZORBAX SB-C_18_)为参比柱,查阅USP网站2个色谱柱数据库提供的相似度值,将实验所用典型色谱柱的实际分离效果与数据库选择性差异的预测结果进行比较,结果见表5。

**表 5:** 典型C_18_柱的实际分离效果与数据库预测结果的比较

Column	F_USP_	F_PQRI_	Practical separation results
ZORBAX SB-C_18_	0	0	all well separated
Capcell Pak MG Ⅲ C_18_	/	12.05	all well separated
Capcell Pak MG Ⅱ C_18_	1.39	7.84	three peaks not well separated
Capcell Pak MG C_18_	1.3	7.92	two peaks not well separated
ZORBAX Extend-C_18_	2.46	12.6	three peaks not well separated
Xbridge C_18_	1.71	12.46	seven peaks not well separated
Xbridge Shield RP C_18_	2.14	23.6	two peaks not well separated
KROMASIL 100-5 C_18_	/	12.11	four peaks not well separated
BDS HYPERSIL C_18_	/	11.85	four peaks not well separated
LUNA C_18_(2)	1.95	12.95	four peaks not well separated
InertSustain AQ-C_18_	/	14.95	four peaks not well separated
INNOVAL C_18_	/	12.76	three peaks not well separated

*F*_USP_: column similarity value in USP databases; *F*_PQIR_: column similarity value in PQIR databases; /: the related columns were not used or unavailable in the corresponding databases; well separated: the peak could at least baseline separated from adjacent peaks (resolution value not less than 1.5); *F*≤3, the selectivity of the two columns is similar and can be regarded as equivalent columns.

USP数据库结果:有7款色谱柱收载于USP数据库中,与ZORBAX SB-C_18_柱的相似度值*F*_USP_全部小于3,然而实际分离效果中4-羟基苯甲酸和甲基氯异噻唑啉酮在5款色谱柱上出现峰重叠或出峰顺序颠倒(见图2),只有ZORBAX Extend-C_18_柱和Xbridge C_18_柱2款色谱柱得到的23种防腐剂的图谱与参比柱的图谱接近,但两款色谱柱都存在部分色谱峰分离度达不到基线分离的现象(见表3)。

PQRI数据库结果:有12款色谱柱收载于PQRI数据库中,查阅与ZORBAX SB-C_18_柱的相似度值*F*_PQRI_,可知12款色谱柱的相似度值均大于3,都不能视为等效色谱柱。尽管与参考色谱柱的相似度值为12.05,但是实际分离效果结果显示,Capcell Pak MG Ⅲ C_18_柱仍能实现对23种组分的良好分离(见图1)。

从现有的实验数据分析,USP网站提供的2种色谱柱选择数据库无法预测出合适的等效色谱柱,对23种防腐剂的液相色谱分析参考价值均较小,这可能与待测组分结构,以及其他色谱条件如流动相pH和柱温等因素有关,进而说明化妆品中多组分分析的复杂性。

### 2.6 应对措施探讨

液相色谱方法容易受到仪器型号、流动相组成及其pH值、色谱柱、柱温和流速等因素影响^[14,15]^。标准方法或规范方法会被不同实验室、不同人员在不同时间和试验条件下使用,可能会发生许多异常或偏差。随着液相色谱方法研究和应用的深入,色谱系统适用性也逐渐受到重视,当检测结果容易受实验条件变化影响时,应当对方法进行合理控制或是指明注意事项。《中国药典》2020年版第四部通则0512高效液相色谱法规定^[16]^:色谱参数除填充剂种类、流动相组分、检测器类型不得改变外,其余如色谱柱内径与长度、填充剂粒径、流动相流速、流动相组分比例、柱温、进样量、检测器的灵敏度等,均可适当调整以满足系统适应性要求。《化妆品安全技术规范》中对液相色谱方法的系统适应性及如何进行色谱参数调整均未做任何说明。方法应用时如果遇到待测组分分离度不佳或杂质峰干扰时,实验人员势必会对方法进行改动,从而造成方法偏移。如果这种更改不加以指导和限制,就会给检测结果的准确性带来很多不可控因素。为解决上述问题,一方面,标准研制单位可以在方法开发初始,通过进行耐用性试验对影响因素做出一定的模拟或预判,以保证方法的可靠性和有效性;另一方面,虽然为体现公正性和避免商业竞争,方法在非必要条件下一般不予公开方法制修订时使用的色谱柱商标等,但是如果测试条件对色谱柱要求苛刻,那么在规定色谱条件时,可以参照食品安全国家标准^[17,18]^细化色谱柱分类和增加描述信息,并注明可以接受变动的范围,或者以标准方法释义^[19]^、编制说明^[20]^或资料性附录等方式,明确色谱柱具体参数,以便于研究人员选择合适的色谱柱,获得最佳的分离效果。

## 3 结论

通过应用《化妆品安全技术规范》中收载的23种防腐剂测定方法,对不同色谱柱分离效果进行分析,并尝试用等效色谱柱选择系统对色谱柱进行评价和预测。实验结果表明:色谱柱是影响化妆品中23种防腐剂液相色谱法测定结果准确性的关键因素,不同品牌C_18_色谱柱的选择性差异明显,但目前仍缺乏能有效预测等效色谱柱的评价机制。有关检验机构在应用规范方法时,应考虑色谱柱选择性差异。建议化妆品标准(规范)管理部门在起草和执行标准(规范)方法时,增加系统适应性和方法耐用性的考察要求,细化色谱柱的分类和系统筛选工作,指导色谱柱的合理选择,减少因色谱柱选择差异导致的结果不准确等问题。如何在现有研究成果的基础上,开发色谱柱的筛选和预测评价体系,进而指导实际样品的分离以适应化妆品基质复杂的特点是下一步研究的重点和难点。
